# Shp2 confers cisplatin resistance in small cell lung cancer via an AKT-mediated increase in CA916798

**DOI:** 10.18632/oncotarget.15641

**Published:** 2017-02-23

**Authors:** Xuemei Yang, Chunlan Tang, Hu Luo, Haijing Wang, Xiangdong Zhou

**Affiliations:** ^1^ Department of Respiratory, Southwest Hospital, Third Military Medical University, Shapingba District, Chongqing 400038, PR China; ^2^ Department of Respiratory, Institute of Surgery Research, Daping Hospital, Third Military Medical University, Yuzhong District, Chongqing 400042, PR China

**Keywords:** small cell lung cancer, cisplatin resistance, SHP2, AKT, CA916798

## Abstract

The tyrosine phosphatase Shp2 is associated with tumorigenesis in small cell lung cancer (SCLC). However, the relationship between Shp2 and resistance to chemotherapy remains unclear. Here, we show that Shp2 plays an important role in inducing resistance to cisplatin-based chemotherapy via the SHP2-AKT-CA916798 pathway. In an SCLC cell line, overexpression of Shp2 induced cisplatin resistance and the increased expression of AKT, pAKT, pmTOR, and CA916798. Conversely, depletion of Shp2 in a cisplatin-resistant cell line via RNA interference increased cisplatin sensitivity and decreased AKT, pAKT, pmTOR, and CA916798 expression levels. Activation of AKT stimulated CA916798 expression and altered the level of Shp2. A mouse xenograft model verified the results obtained from the *in vitro* experiments. In addition, we collected and analyzed clinical SCLC specimens and found that Shp2 levels correlated with CA916798 expression in tumor tissues. Importantly, higher levels of Shp2 or CA916798 were associated with a poorer prognosis in SCLC patients who received chemotherapy. Together, our findings indicate that Shp2 induces cisplatin resistance in SCLC patients via the SHP2-AKT-CA916798 pathway. Therefore, Shp2 and CA916798 may be promising biomarkers for predicting resistance to chemotherapy and may function as targets for enhancing treatments.

## INTRODUCTION

Cisplatin is a platinum-containing first-line drug often used in combination chemotherapy for small cell lung cancer (SCLC). Clinically, patients with SCLC show obvious improvement after initial chemotherapy, but many of them gradually develop cisplatin resistance and subsequent failure in chemotherapy, resulting in reduced five-year survival rates [1, 2]. Currently, the mechanism involved in cisplatin-induced drug resistance in cancer cells is largely unknown.

Shp2 is a cytoplasmic non-receptor-type protein tyrosine phosphatase with a wide array of functions. It is encoded by the PTPN11 gene and contains one protein phosphatase catalytic domain and two Shp2 domains [3]. Biologically, Shp2 is closely associated with cell survival and migration. Shp2 is involved in the development of many tumors, such as leukemia, gastric cancer, and breast cancer. Additionally, Shp2 has been implicated in chemotherapy resistance in breast cancer [4, 5]. Our previous study revealed that Shp2 expression is higher in tumor tissues in lung cancer patients with chemotherapy resistance than in controls, indicating that Shp2 is associated with resistance to lung cancer chemotherapy [6].

Shp2 performs its biological functions by regulating multiple pathways, including the Ras/ERK, JAK-STAT3, and PI3K/AKT pathways [7]. Our previous studies indicated that the expression levels of Shp2 and AKT are elevated in tumor tissues of patients with lung cancer [8, 9]. This study was designed to determine whether Shp2 affects the sensitivity of SCLC to cisplatin, and to explore a possible mechanism by which Shp2 affects cisplatin resistance by altering the levels of AKT and its downstream effector(s). We also examined Shp2-binding partners and Shp2 expression levels in comparison to clinical prognoses in patients with SCLC.

## RESULTS

### Shp2 expression affects the sensitivity of SCLC to cisplatin

**Figure 1:**
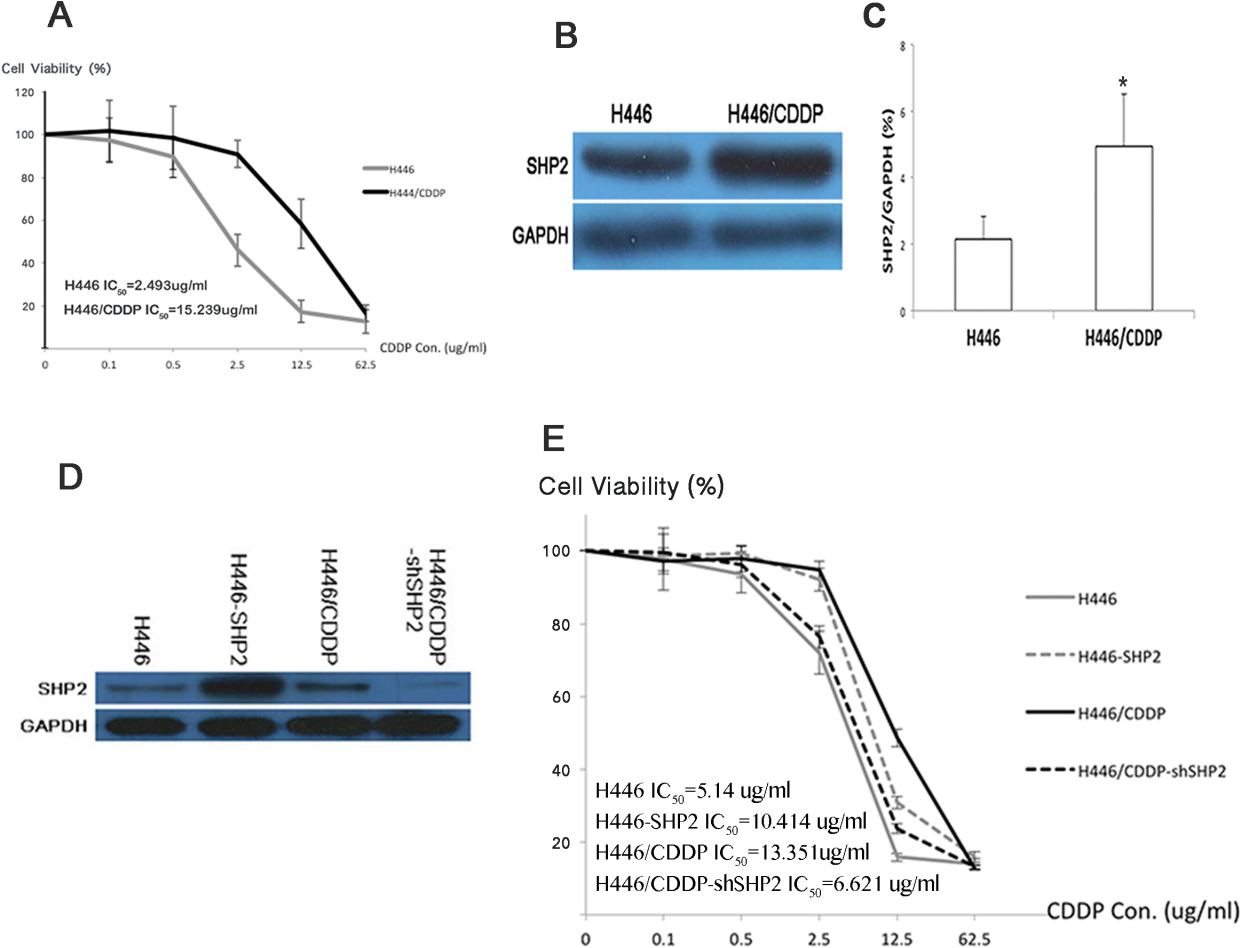
Shp2 affects SCLC's sensitivity to cisplatin **A**. The survival rates of H446 and H446/CDDP cells exposed to various concentrations of cisplatin (CDDP). Each group of cells was seeded into a 96-well plate and cultured in medium containing 0, 0.1, 0.5, 2.5, 12.5, or 62.5 μg/ml cisplatin. Cell survival rates were analyzed 24 hours after the cultures were seeded. **B**. Western blot analysis was used to detect Shp2 protein levels in H446 and H446/CDDP cells. Supernatant was extracted after cells were lysed and incubated with antibodies. Chemiluminescence was used for visualization of the bands. GAPDH was used as a reference. **C**. Densitometric analysis of western blot data. *H446/CDDP compared to H446, P<0.05. **D**. Western blot analysis was used to determine Shp2 protein levels before and after SHP2 expression was altered via overexpression (H446-SHP2) or RNAi (H446/CDDP-shSHP2). GAPDH was used as a reference. **E**. CCK8 assays were used to test viabilities of various cell types treated with various cisplatin concentrations. Each experiment was conducted three times and representative images are shown.

To verify whether Shp2 has an impact on cisplatin (CDDP) sensitivity in SCLC, we treated the H446 (cisplatin-sensitive) and H446/CDDP (cisplatin-resistant) SCLC cell lines with cisplatin to induce stable drug resistance. CCK8 cytotoxicity assays indicated that the IC_50_ of cisplatin in H446 cells was 2.493 μg/ml, while it was 15.239 μg/ml in H446/CDDP cells (Figure 1A). Further analysis by western blot showed that H446/CDDP cells contained a much higher level of Shp2 than did H446 cells (P <0.05) (Figure 1B and 1C). Overexpression of SHP2 in H446 cells caused the IC_50_ of cisplatin to rise from 5.14 μg/ml to 10.414 μg/ml, while shSHP2 RNA interference (RNAi; established by transfection with a short hairpin SHP2 RNAi plasmid) in H446/CDDP cells decreased the IC_50_ of cisplatin from 13.351 μg/ml to 6.621 μg/ml (Figure 1D and 1E). These results indicate that Shp2 expression positively correlates with cisplatin resistance in these SCLC cell lines.

### Shp2 binds to PI3K and CA916798 in cisplatin-resistant cells

**Figure 2:**
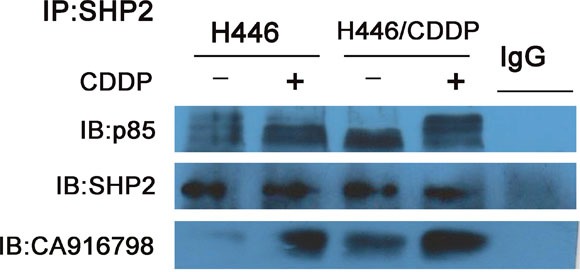
Binding of Shp2 to p85 and CA916798 Cell lysates were incubated with 2 μg/ml of Shp2 antibody and co-immunoprecipitation was performed. Western-blot analysis was used to measure the levels of Shp2, the PI3K p85 subunit, and CA916798 in the precipitated proteins. Each experiment was performed three times and representative images are shown.

We used a co-immunoprecipitation method to precipitate Shp2 and the proteins that bind Shp2. As shown in Figure 2, we detected the quantity of PI3K p85 that bound to Shp2 in SCLC cell lines. Furthermore, in H446 and H446/CDDP cells, Shp2 bound both PI3K p85 and CA916798 - a kind of drug resistance associated protein -before and after stimulation with cisplatin. CA916798 is a drug-resistance associated protein that has previously been shown to affect cisplatin resistance in lung cancer [9, 10]. In the cisplatin-resistant cell line H446/CDDP, an equal amount of Shp2 bound more p85 and CA916798 than did Shp2 in the cisplatin-sensitive cell line H446. Stimulation with cisplatin slightly increased the rate of binding between Shp2 and PI3K. Based on these findings, we sought to determine if CA916798 plays a role in the process by which Shp2 induces cisplatin resistance in SCLC.

### Changes in Shp2 expression affect AKT/mTOR/CA916798 expression

**Figure 3:**
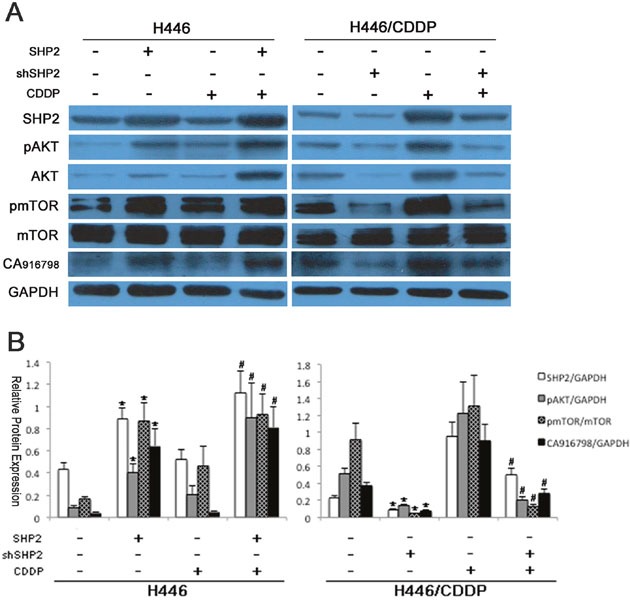
Protein expression before and after Shp2 expression was blocked via RNAi Cells were lysed and proteins extracted. Western blot analysis was used to detect Shp2, AKT, pAKT, mTOR, pmTOR, and CA916798 in each group of cells before and after Shp2 expression was blocked via RNAi. GAPDH was used as a reference. Each experiment was repeated three times. **A**. A representative image. **B**. Results of the western blot analyses. *Comparison of cell protein expression before and after Shp2 was blocked, P<0.05. ^#^Comparison of cell protein expression before and after cisplatin intervention, P<0.05.

To further explore the role of AKT and its downstream signaling pathway in Shp2-induced drug resistance in SCLC, we examined the expression of relevant members of the AKT downstream pathway in SCLC cell lines with induced or inhibited Shp2 expression with or without cisplatin treatment. As shown in Figure 3, when Shp2 expression was increased in H446 cells, the protein levels of AKT, pAKT, pmTOR, and CA916798 were also increased (P<0.05). When cells were treated with cisplatin, the cells with high levels of Shp2 expression showed significantly higher levels of AKT pathway proteins and CA916798 than did the parental cells (P<0.05). In drug-resistant H446/CDDP cells, when Shp2 expression was down-regulated, the expression levels of AKT, pAKT, pmTOR, and CA916798 were also decreased (P<0.05). When the same cells were stimulated with cisplatin, Shp2 expression decreased and the expression of AKT pathway proteins and CA916798 remained reduced (P<0.05). These data suggest that changes in Shp2 expression affect the activity of downstream AKT pathway components and the expression of CA916798.

### The AKT pathway regulates CA916798 expression and cisplatin resistance in SCLC

**Figure 4:**
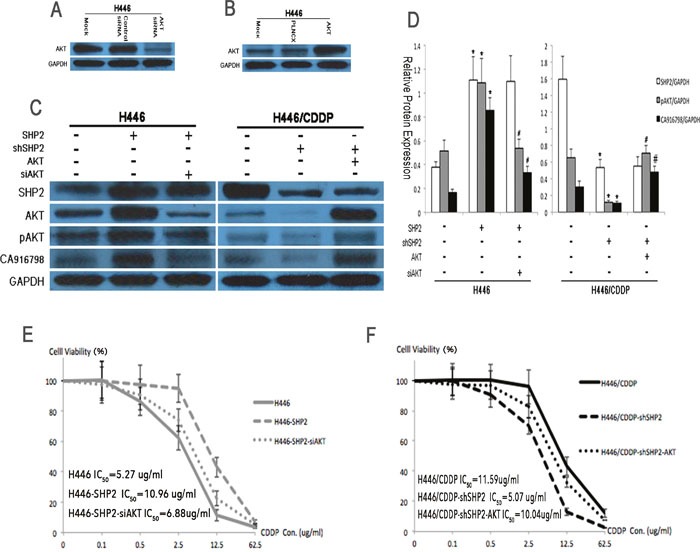
Protein expression levels and cisplatin sensitivity before and after AKT suppression by RNAi **A**. Western blot analysis was used to detect the effects of AKT RNAi on AKT expression levels. The control siRNA was a scrambled RNAi sequence and the mock was an untreated negative control. **B**. Western blot analysis was used to determine the transfection efficiency of AKT shRNA. PLNCX was used as the empty vector and the mock was an untreated negative control. **C** and **D**. Western blot analysis was used to detect Shp2, AKT, pAKT, and CA916798 protein levels before and after the application of Shp2 and AKT RNAi. GAPDH was used as a reference. **E** and **F**. CCK8 assays were used to test cell viability in each treatment group containing various concentrations of cisplatin (CDDP). *Comparison of cells before and after Shp2 was blocked, P<0.05. ^#^Comparison between cells in which Shp2 and AKT were simultaneously blocked and cells in which only Shp2 was blocked, P<0.05. The experiment was repeated three times, and representative images are shown in the figure.

CA916798 is a recently introduced protein in the field of lung cancer drug resistance and the specific mechanisms by which it acts are not yet known. To explore its relationship with the AKT signaling pathway in cisplatin resistance, we simultaneously blocked both SHP2 and AKT expression in H446 cells and then detected the expression of CA916798 and other relevant proteins. In Shp2-overexpressing cells, inhibiting AKT expression reduced the increase in CA916798 expression and cisplatin resistance (Figure 4A, 4C-4E), while in Shp2-knockdown cells, high expression of AKT restored both CA916798 expression and cisplatin resistance to previous levels (Figure 4B, 4C-4D, and 4F). These data indicate that Shp2 regulates CA916798 and cisplatin resistance in SCLC through the AKT pathway and that interfering with the AKT pathway alters CA916798 expression and cisplatin resistance.

### Shp2 affects the sensitivity of SCLC to cisplatin *in vivo*

**Figure 5:**
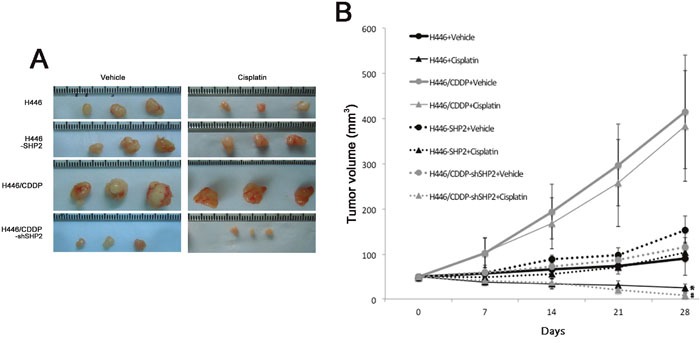
Evaluation of cisplatin sensitivity in tumor cell lines injected into nude mice **A**. Five nude mice in each group were injected subcutaneously with tumor cells, and cisplatin (2 mg/kg/week) or the equivalent volume of vehicle (0.9% NS) was injected into the abdominal cavities of the mice for 4 consecutive weeks. The volumes of the tumors were observed after four weeks. The representative tumor burdens in the groups are shown in the panel. **B**. Cisplatin or vehicle was injected into the abdominal cavities of nude mice and changes in tumor volumes were observed. *Comparison between H446 + cisplatin and H446 + vehicle groups, P<0.05. ^#^Comparison between H446/CDDP-shSHP2 + cisplatin and H446/CDDP-shSHP2 + vehicle groups, P<0.05.

To further validate the results of our *in vitro* experiments, H446, H446-SHP2 (established by transfection of a SHP2 expression plasmid into H446), H446/CDDP, and H446/CDDP-shSHP2 (established by transfection with a SHP2 shRNAi plasmid into H446/CDDP) cells were subcutaneously injected into nude mice, and the sensitivity of each cell line to cisplatin was analyzed. When H446 or H446/CDDP-shSHP2 cells were used, we observed a significant difference in tumor volume between animals injected with cisplatin and those injected with vehicle (saline) (P<0.05). However, there was no significant difference in tumor volume between animals injected with cisplatin and those injected with vehicle when H446-SHP2 or H446/CDDP cells were used (P>0.05) (Figure 5). Additionally, H446 cells, which were sensitive to cisplatin in the *in vitro* experiments, were also sensitive to cisplatin *in vivo* and the H446 cell line that expressed high levels of Shp2 (H446-SHP2) were also resistant to cisplatin *in vivo*. The H446/CDDP cells were resistant to cisplatin both *in vitro* and *in vivo*, but when Shp2 expression was reduced in these cells (H446/CDDP-shSHP2), the cells became sensitive to cisplatin *in vivo*. Therefore, our data confirm that a high level of Shp2 can reduce sensitivity to cisplatin in SCLC and that partially blocking Shp2 in these cell lines can sensitize tumor cells to cisplatin.

### Patients with high levels of Shp2 had high levels of CA916798 and were less sensitive to cisplatin-based chemotherapy

**Figure 6:**
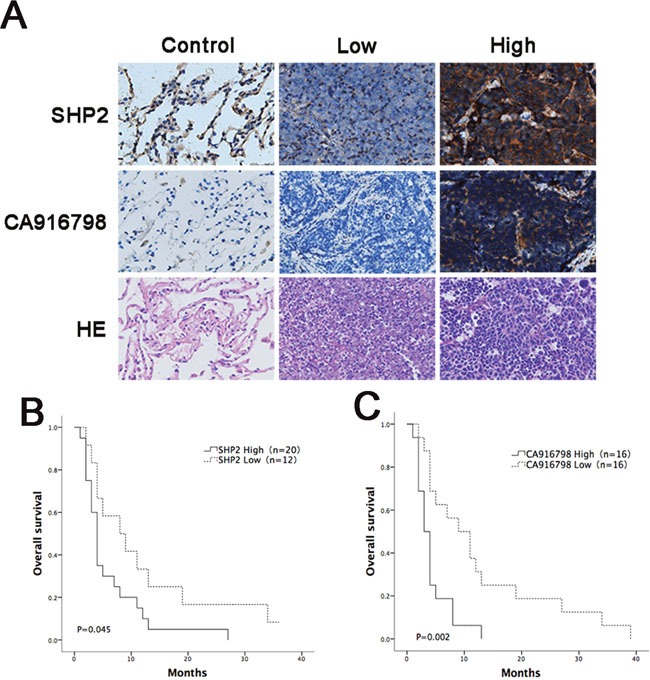
Analysis of survival curves in cancer patients with different expression levels of Shp2 and CA916798 after chemotherapy **A**. Immunohistochemical data for the control group and the groups with high levels of Shp2 or CA916798 or low levels of CA916798 or Shp2 in tumor tissues. Representative images of H&E staining and immunohistochemical staining are shown (×400). **B**. Survival times in patients with different levels of Shp2 in tumor tissues after chemotherapy. **C**. Survival times in patients with different levels of CA916798 in tumor tissues after chemotherapy.

Tumor tissues were isolated from 32 qualified SCLC patients and processed for H&E staining and Shp2 and CA916798 immunohistochemical staining. Four patients with resected benign lesions were selected and nearby benign lung tissue samples were obtained to use as negative controls. Of the 32 SCLC patients, 20 patients had high levels of Shp2. In these patients, the male-to-female ratio was 3:1, their ages averaged 55.950 ± 8.088 years, their initial cycle of chemotherapy was performed 5.15 ± 0.933 times, and the survival time after chemotherapy was 5.340 ± 5.946 months. Twelve of the patients had low levels of Shp2. In these patients, the male-to-female ratio was 2:1, the average age was 54.833±7.941 years, the initial cycle of chemotherapy was performed 5.000 ± 0.953 times, and the survival time after chemotherapy was 11.808 ± 12.105 months. There were no significant differences between these two groups in gender, age, or the number of initial chemotherapy cycles. The results showed that of the 20 patients with high levels of Shp2, 13 patients also had high levels of CA916798 in the pathological samples. Of the 12 patients with low levels of SHP2, only 3 had high levels of CA916798 in the lesion tissues (P<0.05) (Table 1). These data showed that in clinical SCLC patients, tumor tissue levels of Shp2 were positively correlated with CA916798 expression. Overall survival analysis indicated that the patients with high levels of Shp2 had a lower survival rate than those with low levels of Shp2 (P=0.045) and similarly that patients with high levels of CA916798 had a lower survival rate than those with low levels of CA916798 (P=0.002) (Figure 6). These data suggest that in clinical SCLC patients, Shp2 levels were correlated with CA916798 levels in tumor tissues and that patients with high levels of Shp2 and CA916798 had poorer responses and shorter survival periods following cisplatin-based chemotherapy.

**Table 1:** Correlations between the expression levels of Shp2 and CA916798 proteins in patients with SCLC

		Cases (No.)	Shp2		P-value
			Low	High	
CA916798	Low	16	9	7	0.028
	High	16	3	13	

## DISCUSSION

Cisplatin resistance is a major clinical challenge in SCLC chemotherapy. Studies have explored the mechanisms involved in cisplatin resistance and implicate copper transporter 1 (CTR1) [11], multidrug resistance-associated protein 2 (MRP2) [12], and proteins that affect cisplatin resistance by regulating the transport of cisplatin. For example, excision repair cross-complementing gene 1 (ERCC1) affects cellular sensitivity to cisplatin by participating in DNA damage repair [13]. p53, BCL-2, and other apoptosis-related proteins also play important roles in cisplatin resistance [14]. Wu *et al*. suggested that FHIT deficiency may cause cisplatin resistance [15]. These studies have partially explained the mechanisms involved in cisplatin resistance, but measures that have been performed based on that knowledge have not yet overcome cisplatin resistance in clinical tumor tissues. Therefore, we further studied the resistance mechanism in SCLC cell lines and primary tissue samples.

Shp2 is a non-receptor protein tyrosine phosphatase that is widely expressed in various human tissues. It is a key ubiquitous protein that is involved in maintaining the balance between tyrosine phosphorylation and dephosphorylation. In addition to its role as a phosphatase, Shp2 also functions as an important connecting protein in the transduction of multiple signaling pathways [7, 16–18]. Shp2 expression/dysfunction can result in tumorigenesis and metastasis. Moreover, high Shp2 expression in breast, stomach, and lung cancers promotes tumor progression [19–21]. However, some studies have suggested that Shp2 plays an inhibitory role in some types of liver cancers [22, 23]; therefore, its role in tumor developmental processes remains controversial. Thus, we examined the role of Shp2 in cisplatin resistance in SCLC.

Here, we found that Shp2 was associated with cisplatin resistance in SCLC. Increased levels of Shp2 caused the normally cisplatin-sensitive H446 cell line to become resistant, but when Shp2 expression was reduced, H446/CDDP cells that were resistant to cisplatin showed restored sensitivity. Thus, Shp2 expression was positively correlated with sensitivity to cisplatin in SCLC cell lines and Shp2 could induce and even promote resistance to cisplatin. These data also confirmed the notion that Shp2 plays a catalytic role in developmental processes of SCLC.

Shp2 is associated with multiple downstream pathways. Studies exploring the role of Shp2 in signaling pathways have been focused mainly on the MAPK-Erk pathway with very few studies focusing on the function of Shp2 in other pathways. Cisplatin inhibits and kills tumors by inducing apoptosis in tumor cells, and some studies have explored the relationship between the PI3K-AKT-mTOR pathway and cisplatin resistance [24, 25]. Our study found that Shp2 and the PI3K p85 subunit can interact and that expression of AKT signaling pathway proteins were altered when Shp2 levels were altered and vice versa. These data indicate that the AKT signaling pathway may play an important role in the process by which Shp2 affects cisplatin resistance. However, the mechanism by which the pathway exerts its impact via downstream signaling pathways remains unclear.

Co-immunoprecipitation assays showed that Shp2 binds to not only p85, but also CA916798. CA916798 is a newly discovered protein that has been associated with the occurrence and progression of tumors and drug resistance in chemotherapy. It is located in the cytoplasm and nuclei of lung cancer cells. Suppression subtractive hybridization was used to show that CA916798 is differentially expressed between the drug-resistant cell line SPC-A-1/CDDP and its drug-sensitive counterpart SPC-A-1 [26]. The gene encoding CA916798 (GenBank:CA91678.3) is located at 19q13.33 and the molecular weight of its encoded protein is 13.1 kD, which is composed of 117 amino acids, including 47% hydrophobic, 30.8% hydrophilic, 12.8% basic, and 9.4% acidic amino acids. The predicted CA916798 structure shows no significant homology with any other known human proteins, indicating that CA916798 is a novel protein with unknown functions. CA916798 encodes a minor histocompatibility antigen recognized by CD8+ cytotoxic T cells [27] and is an androgen-responsive gene in prostate cancer [28]. However, the mechanism by which CA916798 leads to chemotherapy resistance remains unknown.

Zhou *et al*. reported that CA916798 overexpression increases proliferation rates and reduces apoptosis in H446 cells and enables cisplatin-sensitive H446 cell lines to develop cisplatin resistance [8, 9]. In our experiments, after Shp2 expression was blocked via RNAi, the changes in expression of AKT pathway proteins were positively correlated with changes in CA916798 levels, indicating that CA916798 may be a downstream effector of Shp2 with a role similar to AKT-mTOR. After AKT expression was blocked, CA916798 expression changed accordingly, indicating that CA916798 is downstream of the AKT signaling pathway and that SHP2-AKT-CA916798 is a linkage pathway.

Our data show that high levels of Shp2 induced H446 cells to become resistant to cisplatin. When AKT was inhibited in H446-SHP2 cells that continuously expressed high levels of Shp2, partial resistance to cisplatin was restored. Inhibiting SHP2 expression restored sensitivity to cisplatin in H446/CDDP cells, but overexpressing AKT restored resistance to cisplatin in H446/CDDP-shSHP2 cells. Blocking AKT expression partially offset the impact of changes in Shp2 expression on resistance to cisplatin in SCLC cells. Because changes in the protein levels of Shp2, AKT, and CA916798 were positively correlated with sensitivity to cisplatin, we speculate that the cisplatin sensitivity may be affected by the SHP2-AKT-CA916798 pathway as the mechanism by which Shp2 causes cisplatin resistance in SCLCs.

In our experiments, after Shp2 expression was reduced via shRNA, both total AKT and pAKT expression levels were dramatically altered. These results are inconsistent with those of many other studies that have concluded that the total level of AKT remains unchanged and that only pAKT levels are changed after molecules upstream of AKT are blocked. This is an interesting phenomenon that may be caused by the stable overexpression or down-regulation of Shp2 in these cell lines. The expression levels of pAKT and pmTOR were also slightly increased after cisplatin was added, perhaps because the cells activated anti-apoptotic/anti-death pathways to induce self-rescue when cisplatin induced apoptosis/death. However, the degree to which cisplatin activated apoptosis/death is much greater than that to which the anti-apoptotic/anti-death pathways were activated. Therefore, this effect does not alter the overall effect of cisplatin on SCLC cells.

Animal experiments have shown that H446 cells are cisplatin-sensitive, but that H446-SHP2 cells that have high Shp2 levels lose their sensitivity to cisplatin. H446/CDDP cells are cisplatin-resistant, whereas the H446/CDDP-shSHP2 cell line, in which Shp2 expression is inhibited, exhibited restored sensitivity to cisplatin. The results of our *in vitro* experiments were verified in the *in vivo* experiments. Our clinical results also indicate that CA916798 expression was consistent with Shp2 expression in animals and patients with SCLC. We verified this hypothesis using clinical data that showed that after 4-6 courses of chemotherapy, patients with high levels of Shp2 had shorter survival periods and worse prognoses than those with low levels of Shp2. From a clinical viewpoint, these results confirm that Shp2 plays a major role in resistance to cisplatin-based chemotherapy in SCLC via the AKT-CA916798 pathway. Therefore, Shp2 expression may be an indicator of sensitivity to cisplatin and other platinum-based chemotherapies and can be used to predict prognoses when platinum-containing chemotherapies are applied. Due to the small sample population in this study, additional larger studies will be needed to confirm these findings.

In summary, our research suggests that a high level of Shp2 indicates reduced sensitivity to cisplatin in SCLC and that inhibiting Shp2 expression can restore the sensitivity of SCLC to cisplatin. The effect of Shp2 on the sensitivity of SCLC to cisplatin may function via the PI3K-AKT-mTOR-CA916798 signaling pathway. Blocking Shp2 expression or the transmission of the relevant signaling pathway may enhance the efficacy of platinum-containing chemotherapies on SCLCs and could potentially be used to treat clinically drug-resistant SCLCs.

## MATERIALS AND METHODS

Cell Lines and Culture Conditions: The H446 and H446 drug-resistant (H446/CDDP) cell lines were a gift from Dr. Wang Haijing at the Third Military Medical University(Chongqing, China). Neuron-specific enolase (NSE; a marker for SCLC. Santa Cruz Biotechnolory) expression was confirmed in cell lines by immunofluorescence staining (Supplementary Figure 1). Cells were incubated in Roswell Park Memorial Institute 1640 (RPMI-1640) culture medium (Invitrogen) containing 8% fetal bovine serum (Invitrogen) in a 5% CO_2_ incubator at 37°C. The H446/CDDP cell culture medium was supplemented with 0.2 μg/ml cisplatin (CDDP; Sigma) for 48 hours every two weeks.

Study Participants in the Clinical Experiments: The patients were pathologically diagnosed with SCLC at the Southwest Hospital (Chongqing, China) from 2003 to 2013 and were in the extensive disease stage. The ages of the patients ranged from 18 to 75 and all patients completed 4 to 6 cycles of etoposide + cisplatin chemotherapy. Patients with other malignancies or who received combined radiotherapy or surgery were excluded. All participants had no other uncontrolled serious medical illnesses or infections.

Plasmid Construction and Transfection: A pLKO.1-puro lentiviral vector that contains a puromycin-resistance selection marker was synthesized and constructed by Invitrogen. The transfection process was performed as per the manufacturer's instructions. The Shp2 overexpression plasmid primer sequences were: Forward 5′-aaggatccatgacatcgcggagatggttt-3′ and Reverse 5′-ggctcgagtcatctgaaacttttctgctgttgc-3′. The SHP2 shRNA synthetic sequences were: Forward 5′-gatccaagaaatggagctgtcacccattcaagagatgggtgacagctccatttctttttttg-3′ and Reverse 5′-aattcaaaaaaagaaatggagctgtcacccatctcttgaatgggtgacagctccatttcttg-3′ [29]. The PLNCX-AKT1 and PLNCA-AKT1-K179M plasmids were purchased from Addgene.

Construction of Cell Lines: Puromycin (2 μg/ml. Sigma) was added 24 hours after cell transfection. During subculture of the transfected cells, 2 μg/ml puromycin was added every two weeks to screen the cells and to maintain cell line stability.

CCK8 Cytotoxicity Assays: Cells in the logarithmic phase of growth were selected and seeded in 96-well inoculation plates at 4-5×10^3^ cells/inoculated well. The culture medium was removed after 24 hours and corresponding test wells (3 wells per concentration condition) were filled with culture medium that contained 0, 0.1, 0.5, 2.5, 12.5, and 62.5 μg/ml cisplatin. A blank control group of wells was also set up. The medium in the wells was removed after 48 hours and each well was filled with 200 μl of 20% CCK8 solution (Beyotime Biotechnology) and incubated for 30 minutes at 55°C. The optical density (OD) of the medium was measured at a wavelength of 450 nm. The concentration that inhibited 50% (IC_50_) of the drug's effect was calculated, which indicated the drug resistance index.

Western Blot Analysis: Cells in the logarithmic phase of growth were selected and lysis solution (0.32 M sucrose, 10 mM Tris-HCl [pH 8.0], 1% Triton X-100, 2 mM dithiothreitol, 5 mM EDTA, and 1 mM phenyl methyl sulfonyl fluoride) was added to extract proteins. A total of 50 μg of protein was separated using polyacrylamide gel electrophoresis. Separated proteins were transferred to PVDF membranes. Blots were blocked with 5% non-fat milk in TBST saline and incubated with the appropriate primary antibodies (1:1000-1:3000 dilutions) at 4°C overnight. The p85 and pAKT1 antibodies were purchased from Abcam. The AKT1, mTOR, pmTOR, and GAPDH antibodies were purchased from Santa Cruz Biotechnology. The CA91698 antibody was a gift from Dr. Wang Haijing at the Third Military Medical University. Blots were incubated with secondary antibodies (1:500-1:2000 dilution) at 37°C for 1-2 h. Membranes were treated with chemiluminescence substrates (Millipore) and exposed to medical x-ray film. Protein bands were quantified by densitometric analysis using Image-Pro Plus 6.0 software.

Co-immunoprecipitation: Cells were harvested and immunoprecipitation lysis liquid containing protease inhibitor (Thermo Scientific) was added. Supernatants were removed after centrifugation. IgG or 4 mg anti-Shp2 antibody (Abcam) per 1 mg protein samples were added to the supernatants and the solutions were incubated overnight at 4°C. Protein A Magnetic Beads (Thermo Scientific) were added at 40 μl/ml and the solutions were incubated overnight at 4°C. After centrifugation, the Protein A Magnetic Beads were collected, 15 μl of 2× SDS sample buffer was added, immunoprecipitated proteins were extracted, and western blot analyses were performed.

Histochemistry and immunohistochemical analyses: Tumor tissues were obtained from patients who matched the study conditions and control donors. These tissues were prepared for immunohistochemical staining and the protocol was performed according to the manufacturer's instructions (Beyotime Biotechnology). In the negative control, PBS replaced the primary antibody. Image Pro-Plus 6.0 software was used to perform optical density corrections and optical density measurements in immunohistochemical images. In a 200× magnified visual field, five areas were randomly selected to measure the mean density of 100 positive cells and to determine the amount of positive staining in the sample. A P-value of P<0.05 was used as the standard to judge the critical high and low protein expression values. If the mean density of Shp2-positive cells was ≥0.05, Shp2 was recorded as having high expression, whereas if the mean density was <0.05, Shp2 was recorded as having low expression. If the mean density of CA916798-positive cells was ≥0.03, CA916798 was recorded as having high expression, whereas if the mean density was <0.03, CA916798 was recorded as having low expression.

Animal experiments: Cells in the logarithmic phase of growth were selected and mixed with 0.9% physiological saline to prepare cell suspensions containing 4-6×10^6^ cells/ml. These preparations were injected into the axillary subcutaneous tissues of nude mice (4-5 weeks old, female) at 100 μl/mouse. When the subcutaneous tumors grew to a maximum diameter of 0.5 cm, intraperitoneal injections of cisplatin (2 mg/kg/week) or vehicle (0.9% NS) were administered for four weeks, after which the mice were sacrificed. During this period, the tumor volumes in the mice were measured every two days [tumor volume = (length × width^2^)/2]. Every group has five mice for this study.

Statistical analysis: SPSS 21.0 software was used to analyze the data. One-way analysis of variance was used for comparisons across multiple sets of experimental data, while independent sample t-tests were used for comparisons between two sets of experimental data. The χ^2^ test was used in the relevant analyses to determine differences in the immunohistochemical expression of proteins. A life table analysis was performed to determine overall survival (OS). Differences with a value of P <0.05 were considered to be significant.

## SUPPLEMENTARY MATERIALS FIGURES AND TABLES



## References

[1] Kallianos A, Rapti A, Zarogoulidis P, Tsakiridis K, Mpakas A, Katsikogiannis N, Kougioumtzi I, Li Q, Huang H, Zaric B, Perin B, Courcoutsakis N, Zarogoulidis K (2013). Therapeutic procedure in small cell lung cancer. Journal of thoracic disease.

[2] Sgambato A, Casaluce F, Maione P, Rossi A, Sacco PC, Panzone F, Ciardiello F, Gridelli C (2013). Medical treatment of small cell lung cancer: state of the art and new development. Expert opinion on pharmacotherapy.

[3] Tajan M, de Rocca Serra A, Valet P, Edouard T, Yart A (2015). SHP2 sails from physiology to pathology. European journal of medical genetics.

[4] Zhang J, Zhang F, Niu R (2015). Functions of Shp2 in cancer. Journal of cellular and molecular medicine.

[5] Zhou X, Agazie YM (2009). Molecular mechanism for SHP2 in promoting HER2-induced signaling and transformation. The Journal of biological chemistry.

[6] Tang C, Luo D, Yang H, Wang Q, Zhang R, Liu G, Zhou X (2013). Expression of SHP2 and related markers in non-small cell lung cancer: a tissue microarray study of 80 cases. Applied immunohistochemistry & molecular morphology.

[7] Huang WQ, Lin Q, Zhuang X, Cai LL, Ruan RS, Lu ZX, Tzeng CM (2014). Structure, function, and pathogenesis of SHP2 in developmental disorders and tumorigenesis. Current cancer drug targets.

[8] Wang YL, Zhu BJ, Qi ZZ, Wang HJ, Zhou XD (2013). Akt1 enhances CA916798 expression through mTOR pathway. PloS one.

[9] Wang HJ, Yang HP, Zhou XD, Dai XT, Chen YF, Xiong W (2011). CA916798 regulates multidrug resistance of lung cancer cells. Asian Pacific journal of cancer prevention.

[10] Qi Z, Wang Y, Zhou X (2012). [CA916798 gene participates in cisplatin resistance of human lung adenocarcinoma A549 cells through PI3K/AKT/mTOR pathway]. [Article in Chinese]. Nan Fang Yi Ke Da Xue Xue Bao.

[11] Kuo MT, Fu S, Savaraj N, Chen HH (2012). Role of the human high-affinity copper transporter in copper homeostasis regulation and cisplatin sensitivity in cancer chemotherapy. Cancer research.

[12] Vinette V, Placet M, Arguin G, Gendron FP (2015). Multidrug resistance-associated protein 2 expression is upregulated by adenosine 5〲-triphosphate in colorectal cancer cells and enhances their survival to chemotherapeutic drugs. PloS one.

[13] Jordheim LP, Cros-Perrial E, Matera EL, Bouledrak K, Dumontet C (2014). Expression of domains for protein-protein interaction of nucleotide excision repair proteins modifies cancer cell sensitivity to platinum derivatives and genomic stability. Clinical and experimental pharmacology & physiology.

[14] O'Grady S, Finn SP, Cuffe S, Richard DJ, O'Byrne KJ, Barr MP (2014). The role of DNA repair pathways in cisplatin resistant lung cancer. Cancer treatment reviews.

[15] Wu DW, Lee MC, Hsu NY, Wu TC, Wu JY, Wang YC, Cheng YW, Chen CY, Lee H (2015). FHIT loss confers cisplatin resistance in lung cancer via the AKT/NF-kappaB/Slug-mediated PUMA reduction. Oncogene.

[16] Darian E, Guvench O, Yu B, Qu CK, MacKerell AD (2011). Structural mechanism associated with domain opening in gain-of-function mutations in SHP2 phosphatase. Proteins.

[17] Broxmeyer HE, Etienne-Julan M, Gotoh A, Braun SE, Lu L, Cooper S, Feng GS, Li XJ, Chan RJ (2013). Hematopoietic colony formation from human growth factor-dependent TF1 cells and human cord blood myeloid progenitor cells depends on SHP2 phosphatase function. Stem cells and development.

[18] Haider UG, Roos TU, Kontaridis MI, Neel BG, Sorescu D, Griendling KK, Vollmar AM, Dirsch VM (2005). Resveratrol inhibits angiotensin II- and epidermal growth factor-mediated Akt activation: role of Gab1 and Shp2. Molecular pharmacology.

[19] Muenst S, Obermann EC, Gao F, Oertli D, Viehl CT, Weber WP, Fleming T, Gillanders WE, Soysal SD (2013). Src homology phosphotyrosyl phosphatase-2 expression is an independent negative prognostic factor in human breast cancer. Histopathology.

[20] Schneeberger VE, Luetteke N, Ren Y, Berns H, Chen L, Foroutan P, Martinez GV, Haura EB, Chen J, Coppola D, Wu J (2014). SHP2E76K mutant promotes lung tumorigenesis in transgenic mice. Carcinogenesis.

[21] Miyamoto D, Miyamoto M, Takahashi A, Yomogita Y, Higashi H, Kondo S, Hatakeyama M (2008). Isolation of a distinct class of gain-of-function SHP-2 mutants with oncogenic RAS-like transforming activity from solid tumors. Oncogene.

[22] Bard-Chapeau EA, Li S, Ding J, Zhang SS, Zhu HH, Princen F, Fang DD, Han T, Bailly-Maitre B, Poli V, Varki NM, Wang H, Feng GS (2011). Ptpn11/Shp2 acts as a tumor suppressor in hepatocellular carcinogenesis. Cancer cell.

[23] Jiang C, Hu F, Tai Y, Du J, Mao B, Yuan Z, Wang Y, Wei L (2012). The tumor suppressor role of Src homology phosphotyrosine phosphatase 2 in hepatocellular carcinoma. Journal of cancer research and clinical oncology.

[24] Liu Z, Zhu G, Getzenberg RH, Veltri RW (2015). The upregulation of PI3K/Akt and MAP kinase pathways is associated with resistance of microtubule-targeting drugs in prostate cancer. Journal of cellular biochemistry.

[25] Park J, Ko YS, Yoon J, Kim MA, Park JW, Kim WH, Choi Y, Kim JH, Cheon Y, Lee BL (2014). The forkhead transcription factor FOXO1 mediates cisplatin resistance in gastric cancer cells by activating phosphoinositide 3-kinase/Akt pathway. Gastric cancer.

[26] Chen J, Qian GS, Huang GJ, XIiong W, Li J (2001). Suppression subtractive hybridization identified genes differentially expressed in a multidrug resistance cell line of human lung adenocarcinoma. Chin J Cancer.

[27] Tykodi SS, Fujii N, Vigneron N, Lu SM, Mito JK, Miranda MX, Chou J, Voong LN, Thompson JA, Sandmaier BM, Cresswell P, Van den Eynde B, Riddell SR (2008). C19orf48 encodes a minor histocompatibility antigen recognized by CD8+ cytotoxic T cells from renal cell carcinoma patients. Clin Cancer Res.

[28] Romanuik TL, Wang G, Holt RA, Jones SJ, Marra MA, Sadar MD (2009). Identification of novel androgen-responsive genes by sequencing of LongSAGE libraries. BMC Genomics.

[29] Zhou XD, Agazie YM (2008). Inhibition of SHP2 leads to mesenchymal to epithelial transition in breast cancer cells. Cell death and differentiation.

