# Supplementary material for: Shp2 confers cisplatin resistance in small cell lung cancer via an AKT-mediated increase in CA916798
Source: Oncotarget. 2017 Feb 23;8(14):23664–74. doi: 10.18632/oncotarget.15641 (PMC5410335; doi:10.18632/oncotarget.15641)
Supplement: Supplementary file 1 [file oncotarget-08-23664-s001.pdf]

Shp2 confers cisplatin resistance in small cell lung cancer via an AKT-mediated increase in CA916798

SUPPLEMENTARY FIGURE

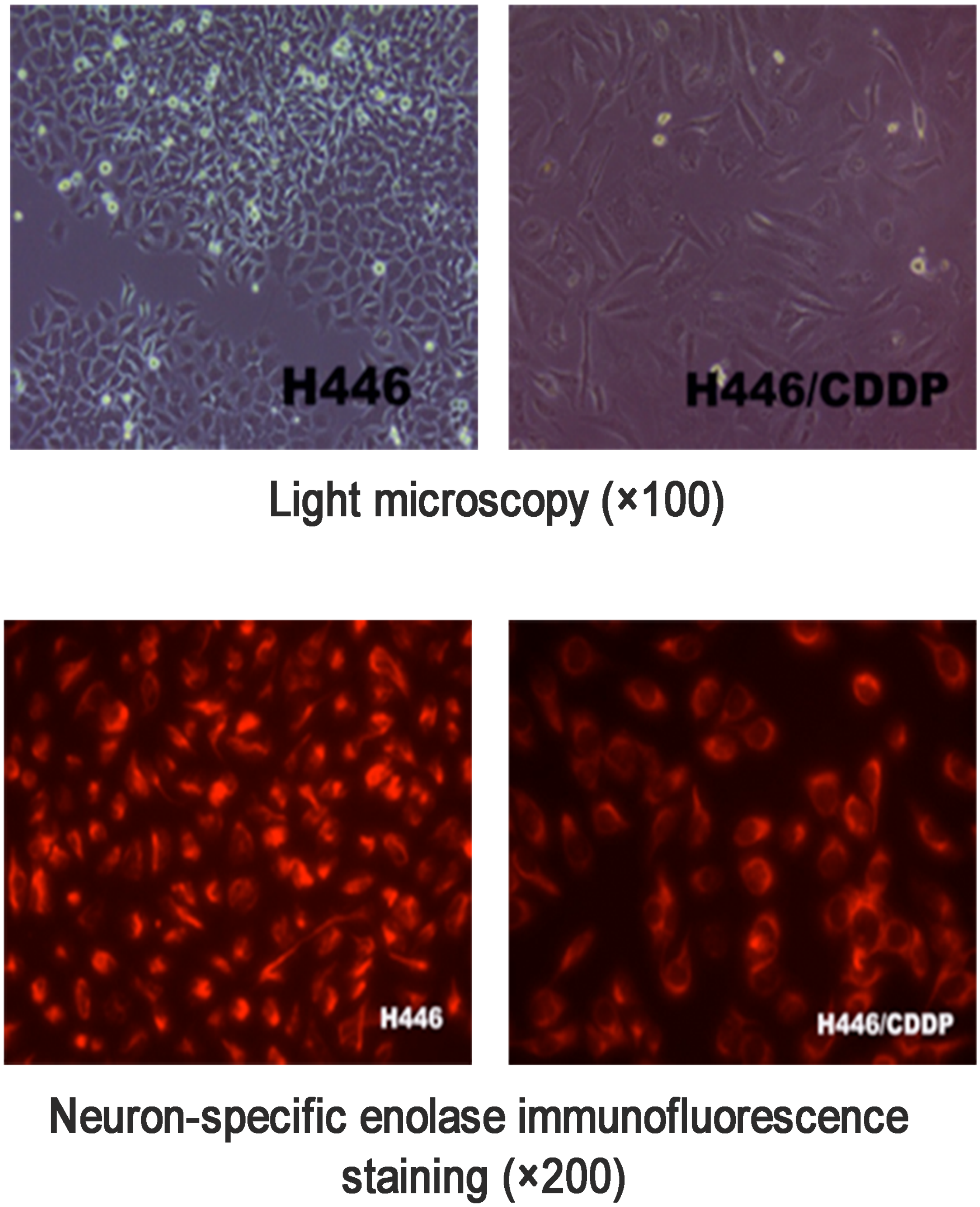

Supplementary Figure 1: Identification of H446 and H446/CDDP cells. A-B. Cell morphology of H446 and H446/CDDP (× 100). **C-D.** NSE immunofluorescence staining of H446 and H446/CDDP cell lines (× 200). SCLC cells show positive expression of NSE (Red fluorescence.
